# Olive Pomace Phenolic Compounds Stability and Safety Evaluation: From Raw Material to Future Ophthalmic Applications

**DOI:** 10.3390/molecules26196002

**Published:** 2021-10-02

**Authors:** Nikolaos Katsinas, Amalia Enríquez-de-Salamanca, Andreia Bento da Silva, Maria Rosário Bronze, Soraya Rodríguez-Rojo

**Affiliations:** 1 Research Institute on Bioeconomy (BioEcoUVa), High Pressure Processes Group, School of Engineering, University of Valladolid (UVa), Dr. Mergelina Street, 47011 Valladolid, Spain; nkatsinas@ioba.med.uva.es; 2 Institute of Applied Ophthalmobiology (IOBA), Campus Miguel Delibes, University of Valladolid (UVa), Paseo de Belén 17, 47011 Valladolid, Spain; amalia@ioba.med.uva.es; 3 Biomedical Research Networking Center in Bioengineering, Biomaterials and Nanomedicine (CIBER-BBN), Av. Monforte de Lemos, 3–5, 28029 Madrid, Spain; 4 Instituto de Investigação do Medicamento (iMed.ULisboa), Faculdade de Farmácia, Universidade de Lisboa (FFULisboa), Av. Prof. Gama Pinto, 1649-019 Lisbon, Portugal; abentosilva@ff.ulisboa.pt (A.B.d.S.); mrbronze@ff.ulisboa.pt (M.R.B.); 5 Departamento de Ciências Farmacêuticas e do Medicamento (DCFM), Faculdade de Farmácia da Universidade de Lisboa (FFUL), Av. das Forças Armadas, 1649-003 Lisboa, Portugal; 6 Faculdade de Ciências e Tecnologia da Universidade Nova de Lisboa (FCT NOVA), Largo da Torre, 2829-516 Caparica, Portugal; 7 Instituto de Biologia Experimental e Tecnológica (iBET), Apartado 12, 2780-901 Oeiras, Portugal; 8 Instituto de Tecnologia Química e Biológica da Universidade Nova de Lisboa (ITQB NOVA), Av. da República, 2780-157 Oeiras, Portugal

**Keywords:** olive pomace, phenolic extracts, oleuropein, hydroxytyrosol, storage stability, genotoxicity assay

## Abstract

Nowadays, increasing interest in olive pomace (OP) valorization aims to improve olive’s industry sustainability. Interestingly, several studies propose a high-value application for OP extracts containing its main phenolic compounds, hydroxytyrosol and oleuropein, as therapy for ocular surface diseases. In this work, the stability and accessibility of OP total phenolic and flavonoid content, main representative compounds, and antioxidant activity were assessed under different pretreatment conditions. Among them, lyophilization and supercritical CO_2_ extraction were found to increase significantly most responses measured in the produced extracts. Two selected extracts (CONV and OPT3) were obtained by different techniques (conventional and pressurized liquid extraction); Their aqueous solutions were characterized by HPLC-DAD-MS/MS. Additionally, their safety and stability were evaluated according to EMA requirements towards their approval as ophthalmic products: their genotoxic effect on ocular surface cells and their 6-months storage stability at 4 different temperature/moisture conditions (CPMP/ICH/2736/99), together with pure hydroxytyrosol and oleuropein solutions. The concentration of hydroxytyrosol and oleuropein in pure or extract solutions was tracked, and possible degradation products were putatively identified by HPLC-DAD-MS/MS. Hydroxytyrosol and oleuropein had different stability as standard or extract solutions, with oleuropein also showing different degradation profile. All compounds/extracts were safe for ophthalmic use at the concentrations tested.

## 1. Introduction

The annual cultivation of olive groves reaches a surface of 10.6 million hectares at a global level (data 2019). Within the last decade, olive oil production worldwide has increased by 20%, being approximately 20,069,835 tons per year for the period 2010–2019. According to FAOSTAT, 72% of the olive oil is produced by the Mediterranean countries of Europe [1]. This fact can be explained by the well-known importance of the Mediterranean diet for human health, with olive oil being its principal fat ingredient [2]. The numerous health benefits of olive oil are mostly related to its antioxidant fatty acids and phenolic compounds [3]. Among the latest, hydroxytyrosol (HT) and oleuropein (OL) are the major representatives with several biological activities reported, such as antioxidant, anti-inflammatory, antimicrobial, anticancer, antiatherogenic, and cardioprotective [4]. During olive oil and table olive production, huge amounts of by-products are produced, such as the olive pomace (OP), the mill wastewaters, the stones, and the leaves. The OP is the main by-product generated and is a mixture of olive fruits after the recovery of olive oil, together with vegetation waters and stones [5]. Traditionally, the un-treated OP is used to extract the so-called “OP oil”, and afterward, it is burnt or discarded into the soil [2,5,6]. Its high organic load and phenolic content, along with its phytotoxic properties, make the OP a potential source of soil, water, or air pollution [2,7,8].

Several alternative applications have been studied for the OP in a biorefinery framework, such as biofuel production and agronomic uses, among others [2,5]. Its valorization as a source of valuable bio-active phenolic compounds is an emerging issue, as the OP is rich in phenolic compounds, including HT and OL [9,10]. Numerous studies have valorized the OP for the recovery of these molecules using different extraction techniques, varying from conventional solid–liquid extraction [11] to intensified and environmentally friendly processes such as microwaves and ultrasounds [12,13,14,15]. Recently, our group [16] proposed a combination of two sustainable techniques (supercritical carbon dioxide extraction—scCO_2_ and pressurized liquid extraction—PLE) for the selective and optimal recovery of these compounds from the OP. Furthermore, in another study [17], we also demonstrated the strong antioxidant and anti-inflammatory activity of two OP extracts, together with the pure HT and OL (alone or in combination) on human corneal and conjunctival epithelial cells. Di Mauro et al. [18] have also proved the same activities on rabbit corneal cells for polyphenolic fractions from olive mill wastewaters with HT as the major component. Several diseases of the ocular surface include oxidative stress and inflammation in their pathophysiology, such as dry eye and ocular allergy [19,20,21]. The oxidative damage can also stimulate ocular inflammation [20], while it is involved in conjunctivochalasis [22] and keratoconjunctivitis [23]. Therefore, the use of HT- or OL-rich extracts derived from OP, as well as pure OL and HT, could be a potential treatment for oxidative and inflammatory-related diseases of the ocular surface.

For the approval of these compounds and extracts as an ophthalmic product, it is necessary to previously evaluate their stability and safety. Each olive phenolic compound class demonstrates different stability [24]. For example, secoiridoids such as OL are more unstable and thus can be easily hydrolyzed to simple phenols such as HT [16,25,26]. It is also true that the richness of the material in the phenolic compounds of interest can vary depending on geographical, climatic, and varietal issues [27,28]. However, it is also affected by other factors that can be controlled, such as drying and storage conditions. The effect of long-term storage (varying from 8 to 18 months) on the stability or oxidation of the virgin olive oil phenolic compounds has been widely studied at several temperatures (from 5 to 50 °C) [24,29,30]. More recently, ^1^H-NMR metabolomic fingerprinting of the same material after light (500 lux) and temperature (25, 30, and 35 °C) stress has been performed for 12 or 24 months [31]. It was shown that the olive phenolic compounds, especially secoiridoid derivatives, have low thermal and oxidative stability. Hence, changes in phenolic composition occur during long-term storage (reduction of secoiridoids accompanied by an increase of specific simple phenols), together with glyceride degradation and isomerization. For extracts derived from olive leaves, different drying procedures and storage temperatures have been applied either in liquid hydroalcoholic (80% *v/v* EtOH) or solid (dried) form for a short storage period of 4 weeks [32]. A significant reduction of their total phenolic content (TPC) and antioxidant activity (AA) was observed after drying. However, storage did not affect any of the responses measured regardless of the storage temperature or the extract form (liquid or solid). In addition, Di Mauro et al. [18] proved that the HT percentage of an ophthalmic hydrogel comprising a polyphenolic fraction derived from olive mill wastewaters can remain stable for 3 months at 25 °C. However, to our knowledge, a study about the stability of the OP phenolic compounds under different conditions has not yet been performed either for the raw material or for aqueous solutions of pure OP compounds or extracts.

The mandatory tests for the approval and release of a pharmaceutical product or drug substance in the market include long-term storage stability and in vitro or in vivo genotoxicity assays. The European Medicines Agency (EMA) guidance CPMP/ICH/2736/99 determines the different thermal and moisture storage conditions in which a pharmaceutical product should remain stable, covering storage, shipment, and subsequent use of the product [33]. Depending on the intended storage condition of the future product, long-term, accelerated, and intermediate storage conditions are described. Usually, long-term storage should cover a minimum of 12 months’ duration, while for intermediate or accelerated studies, a period of 6 months is proposed. However, no degradation protocols have been set for OP extract or OL, while no composition standards exist for the OP extract or powder. For HT, its concentration in the olive oil has been studied during long-term degradation, while impurities have been identified generated by its semi-synthesis or fermentation solution [34,35]. For this reason, authorization as a “novel food ingredient” has been obtained for this molecule [36]. However, none of the protocols have been established for aqueous HT solutions. Furthermore, in the British, European, and United States Pharmacopoeias, there are monographs only for olive leaf powder and dry extracts, describing the standards with which they should comply in terms of composition (content in OL) and contaminants (microorganisms) [37,38,39]. Regarding the mandatory tests for genotoxicity, the ICH guideline S2 (R1) by EMA determines the different assays acceptable for screening possible genetic damage, to predict and avoid potential human risks [40]. Among them, the comet assay is proposed as a reliable and common technique to detect the in vitro genotoxicity of a compound, detecting various types of DNA damage in individual cells [40,41]. For HT, all genotoxicity studies established so far are based on microorganisms and not human cells, while they all concern its oral use and not its topical ophthalmic application [34].

The objective of this work was to evaluate HT, OL, or OP extracts as a future ophthalmic pharmaceutical product, contributing to the valorization of an agro-industrial by-product potentially hazardous for the environment. The effect of storage conditions of the raw material on the stability of its major phenolic compounds has been studied, together with the long-term stability of aqueous solutions of OL, HT, and two different dried OP extracts at four different temperatures (T) and relative humidity (RH) storage conditions (based on the guidance CPMP/ICH/2736/99). Degradation by-products of olive phenolic compounds have been putatively identified by HPLC-DAD-MS/MS and the degradation profile between the extracts and the pure compounds was compared. Furthermore, the in vitro genotoxicity (comet assay) of the solutions has been evaluated on human corneal and conjunctival epithelial cells, to ensure that the possible future drug substances comply with the safety guidelines.

## 2. Results and Discussion

### 2.1. Effect of Pretreatment Conditions on the Material

To screen the stability of major phenolic compounds in the raw material—OP—different pretreatment conditions were tested: fresh, de-frozen, freeze-dried, and dried. The material was subjected to extraction directly after the pretreatment took place, using the same conventional solid–liquid phenolic procedure. It was evaluated in terms of richness in TPC, total flavonoid content (TFC), OL, oleacein (OLC), HT, and tyrosol (TY), as well as extraction yield (EY) and AA. Table 1 presents the results obtained for each of the tests performed for each condition. Furthermore, analysis of variances (ANOVA) was performed for each response among the different pretreatment conditions, to highlight the statistical significance of the results (*p*-values presented in Table S1, Supplementary Material).

According to the results, the pretreatment of the raw material can highly affect the stability of the main compounds. The freeze-dried material demonstrated the highest stability for all measured phenolic compounds, except OL. In fact, freeze-drying has been proved to be a cell disruption method [42,43]. By freezing and subsequent sublimation of water, the cell walls are harmed, increasing the recovery of the compounds of interest [44] and explaining why most of the responses measured are increased by this method. On the other hand, the dried OP demonstrated the lowest stability for all responses measured.

Compared to the fresh OP, the de-frozen had significantly higher TFC (ca. 36%), while the freeze-dried had 40% more TFC and 22% stronger AA. An increase of 8% for the AA was also observed for the de-frozen. However, it was not considered statistically significant. Between the freeze-dried and de-frozen OP, the AA was significantly higher in the first case. In terms of TPC, the variations observed among the different pretreatment conditions were not considered statistically significant. Regarding the extract richness in the compounds of interest, HT and TY were significantly higher in the freeze-dried material (90% more HT compared to fresh, while 46% more TY compared to the de-frozen). A statistically significant increase of the dry extract (DE) richness in OLC by 224% compared to the fresh and by 230% compared to the de-frozen was also observed after lyophilization. However, OL demonstrated a remarkable decrease in the freeze-dried OP (by 66% compared to the de-frozen and by 43% compared to the fresh), while it was significantly increased in the de-frozen material compared to the fresh (by 68%). As it can be observed, the reduction of OL content is accompanied by a proportional increase of OLC richness. This can be explained by possible biotransformation from OL to OLC during the drying process. Sarikaki et al. [45] has already proposed a mechanism for this conversion. This would include cleavage of the methyl ester, followed by a loss of the sugar and an opening of the secologanoside ring, leading to a *seco*-dialdehyde derivative, which is susceptible to decarboxylation. Regarding the EY obtained, it was also significantly lower after lyophilization (ca. 30% vs. fresh OP), while there was no remarkable variation among the rest of the pretreatment conditions. For the dried OP, except TPC and EY that did not demonstrate any significant differences, most of the responses measured were much lower. Particularly, TY and TFC showed a significant decrease in comparison to any of the pretreatment conditions tested, while HT and AA were reduced compared to the freeze-dried and the de-frozen OP. Additionally, OLC and OL were significantly lower compared to the freeze-dried or fresh and the de-frozen material, respectively.

Our findings agree with those previously reported in the bibliography. Jiao et al. [46] demonstrated that freeze–thaw pretreatment up to twofold increases the recovery of natural antioxidants, with the highest obtention yields after thawing material storage at −20 °C. This can be explained by the large ice crystals formed, preventing insoluble components from bounding with the compounds of interest. Additionally, freeze–thaw is a cell disruption method, modifying the cellular structure and increasing the permeability of the cell walls or membranes. Thus, demonstrating higher phenolic availability [47]. According to Zorić et al. [48], drying the raw material can improve its conservation in terms of phenolic compounds, flavonoids, and antioxidant activity. Regarding the drying process, Lang et al. [49] proved that an increase in the temperature decreases the TPC and the TFC of the raw material.

Additionally, it is true that with lyophilization the lowest EY was obtained. However, a low EY can benefit the extract richness in the selected compounds, contributing to its final bioactivity. Since our objective was the highest AA and phenolic content, the freeze-dried OP has been selected among the pretreatment conditions tested.

### 2.2. Effect of the Defatting Pretreatment Step on the Phenolic Content and Profile of OP Extracts

The OP is composed of a fraction of residual oil and lipophilic components [5,6] (see 3.1. for plant material characterization). The ocular surface consists of mostly water-like tissues, such as tear film and aqueous humor [50,51]. Thus, to achieve good diffusion and of the drug in the ocular tissues, water-based formulations should be designed. Additionally, oil-based drops can cause ocular burning, itching, irritation, and blurry vision [52,53]. Therefore, a defatting step prior to the phenolic extract was considered necessary to remove any residual oil from the raw material considering its future application.

For the defatting process, two different methods were performed using freeze-dried OP: one using *n*-hexane and one using scCO_2_. As previously described [16], the oil obtained by the two methods, expressed as percentage of the dry basis, was similar. However, in the case of scCO_2_, toxic organic solvents are avoided, as CO_2_ is cleaner and non-toxic, being a sustainable and not expensive technique at the industrial level [54]. The effect of these two methods on the stability of the responses (TPC, TFC, OL, OLC, HT, TY, EY, and AA) measured has been screened and compared to the reference material (freeze-dried OP with no further pretreatment). The defatted freeze-dried OP samples were subjected to the same conventional solid–liquid phenolic extraction and the obtained extracts were evaluated as presented in Table 2. The *p*-values of the ANOVA analyses performed for each response comparing the two defatting methods between them and with the reference extract (derived by non-defatted OP) are included in Table S2 (Supplementary Material).

According to the results, most of the responses remained stable in both cases. In particular, for the extract derived from *n*-hexane-defatted OP, none of the responses showed any significant variation compared to the reference extract. However, in the case of scCO_2_, the phenolic extract obtained had significantly higher TPC than the reference one (ca. 37%). scCO_2_ displays gas-like transport properties that improve its penetration in the vegetal matrix, while the depressurization step of solvent removal can cause cell disruption [54]. This could explain the higher extraction yield mainly of reducing compounds according to TPC assay, namely phenolic compounds. For the rest of the responses, the variations observed were not considered statistically significant and both defatting processes were similar as well. Thus, it can be said that a defatting step does not affect the phenolic composition of the extract produced afterward.

Furthermore, the defatting pretreatment allows the recovery of the residual oil of the raw material, the so-called “OP oil”, which is already available in the market [5,6]. Moreover, the extraction using scCO_2_ as solvent is a sustainable technique, providing a residual-free extraction product and allowing to obtain an improved quality OP oil compared to the traditional hexane process, as no further refinement is needed [54,55].

### 2.3. Effect of Extract Drying on Phenolic Retention

To compare the retention of the phenolic compounds before and after drying, one part of an extract was kept liquid and another one was dried. Regarding the drying process, samples were analyzed in two sequential steps: after ethanol (EtOH) evaporation (step 1—extract still liquid) and after freeze-drying of the extract (step 2—solid extract). Results are presented in Table 3, while the *p*-values of the ANOVA analyses performed for each response are included in Table S3 (Supplementary Material).

It can be observed that the drying process can highly affect the retention of most of the measured responses of the obtained extract. In particular, the AA and the TPC richness of the extract were significantly reduced by 45% and 47%, respectively; similarly, the HT and the TY richness by 41% and 42%, respectively. However, the reduction of TFC and OLC was not considered statistically significant, while in the case of OL, a significant increase of 69% was observed. Hence, depending on the compound, the drying process can selectively affect its retention. It can be said that for labile and more unstable compounds, such as OL and OLC, extract drying can act beneficially. However, for simple phenols (such as HT and TY), the extract drying leads to the loss of almost half of their quantity. The Folin–Ciocalteu method has been considered by many authors as an antioxidant assay and not a quantification method, as it is also based on electron transfer and its results correlate well with the results obtained by AA measurement assays, such as the oxygen radical absorbance capacity (ORAC) [56,57,58]. In our case, TPC and AA were both decreased similarly, following the bibliography. It is important also to highlight that all changes occur during the step of the sublimation of the water of the freeze-drying, as no statistically significant differences were observed between reference and step 1. Therefore, the evaporation of EtOH does not affect the retention of any of the responses measured.

### 2.4. Evaluation for Ophthalmic Applications of Aqueous Solutions of Selected OP Extracts, OL, and HT

#### 2.4.1. Selection of the OP Extracts

Based on the pretreatment results, the extract produced by conventional solid–liquid extraction from freeze-dried OP (conventional OP extract—CONV) was selected as reference extract. Its selection was also based on its good antioxidant and anti-inflammatory activity on human corneal epithelial (HCE) and immortalized human conjunctival epithelial (IM-ConjEpi) cells [17]. Then, to improve this extract, not only by increasing its richness in phenolic compounds but also by avoiding the presence of oily compounds potentially irritating the ocular surface [52,53], the OPT3 extract was selected. This extract was produced by PLE extraction using freeze-dried OP defatted with scCO_2_ extraction. The operating conditions were optimized in a previous study [16] in comparison to the conventional solid–liquid extraction using the same raw material. Three different optimal extracts were produced by this system, being the OPT3 the optimized extract with the strongest antioxidant and anti-inflammatory activity on HCE and IM-ConjEpi cells [17].

As already mentioned, the inclusion of the defatting step by scCO_2_ extraction in the production of the extract allows the recovery of two different products: first, the so-called “OP oil” with a cleaner process compared to the conventional extraction with hexane; and second, an aqueous-based phenolic extract at the concentrations required for the ocular application. Thus, the CONV and the OPT3 were selected for further stability and safety evaluation, together with the pure HT and OL, as main phenolic compounds. Before this, a characterization by HPLC-DAD-MS/MS of both extracts was considered necessary, to putatively identify the phenolic compounds present and to compare the phenolic profiles.

#### 2.4.2. HPLC-DAD-MS/MS Phenolic Characterization of Aqueous Solutions of CONV and OPT3

**Figure 1:**
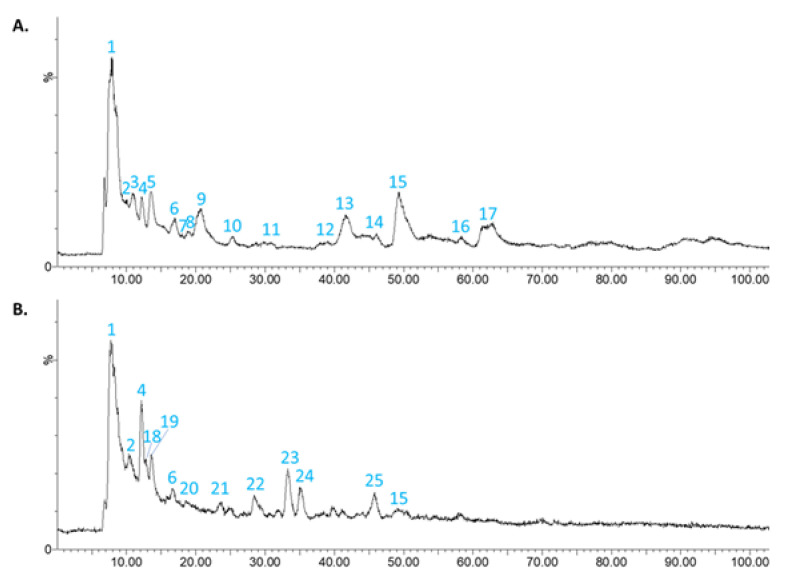
Scan chromatogram in electrospray ionization source in negative mode (ESI−) for the conventional (CONV) (**A**) and the optimized (OPT3) (**B**) olive pomace (OP) dried extracts in aqueous solution.

The HPLC-DAD-MS/MS analysis was performed for aqueous solutions of 5 mg/mL of CONV and OPT3 dried extracts. Figure 1 presents the mass chromatogram of CONV (Figure 1A) and OPT3 (Figure 1B). Table 4 includes the phenolic compounds putatively identified for both extracts. Apart from OL, HT, and TY, 12 compounds were putatively identified in CONV solution, and 6 in OPT3 solution.

As it can be observed, the composition of the two extracts is different, although they were obtained from the same raw material. CONV extract contains more complex molecules, such as secoridoids and secoridoid glucosides, while OPT3 comprises mostly smaller compounds, such as simple phenols and iridoids. This can be explained by the different extraction procedures and conditions applied for the obtention of each extract. CONV was extracted at a low T (70.0 °C) and a medium percentage of ethanol in water (%EtOH) (50.0%), while OPT3 was obtained at high T (184.0 °C) and %EtOH (90.0%). The extraction T can highly affect the type of compounds recovered from the plant material, as thermally labile substances such as secoiridoids can be easily hydrolyzed by applying high T during the extraction process. For example, it has already been proved that OL can be hydrolyzed to HT at high T [16,25,26]. The %EtOH can also affect the compounds obtained in an extract. For example, OL, TY, and HT are better obtained in high %EtOH, due to solubility reasons [16,62,63]. However, the absolute absence of OL in OPT3 can be explained by the high T applied, leading to its complete decomposition.

#### 2.4.3. In Vitro Genotoxicity

According to the ICH guideline S2 (R1), comet assay evaluates the cell DNA damage under a neutral or alkaline electrophoretic field. This leads to the separation between intact and damaged cellular DNA, creating the typical “comet tail” visible from a microscope. By measuring this tail, the extent of the DNA damage can be estimated [41,64]. In this study, we applied alkaline electrophoresis, because it is more sensitive and can detect smaller amounts of DNA damages, including single and double-stranded DNA breaks, alkali labile DNA adducts, and most oxidative DNA damage [65].

Two different human ocular surface cell lines were selected for this assay: HCE and the IM-ConjEpi. The two selected OP extracts (CONV and OPT3) were tested for their in vitro genotoxicity on both cell lines, together with the two principal pure olive compounds OL and HT, alone or in combination (OL+HT). For each compound or extract, the maximum allowable concentration was selected to be tested, based on the results of their in vitro cytotoxicity on the same cell lines, as previously described [17]. Hence, the cells were exposed for 24 h to aqueous solutions of 300 μM of OL, 100 μM of HT, 5 μM + 50 μM of OL+HT, 0.4 mg/mL of OPT3, and 0.8 mg/mL of CONV.

**Figure 2:**
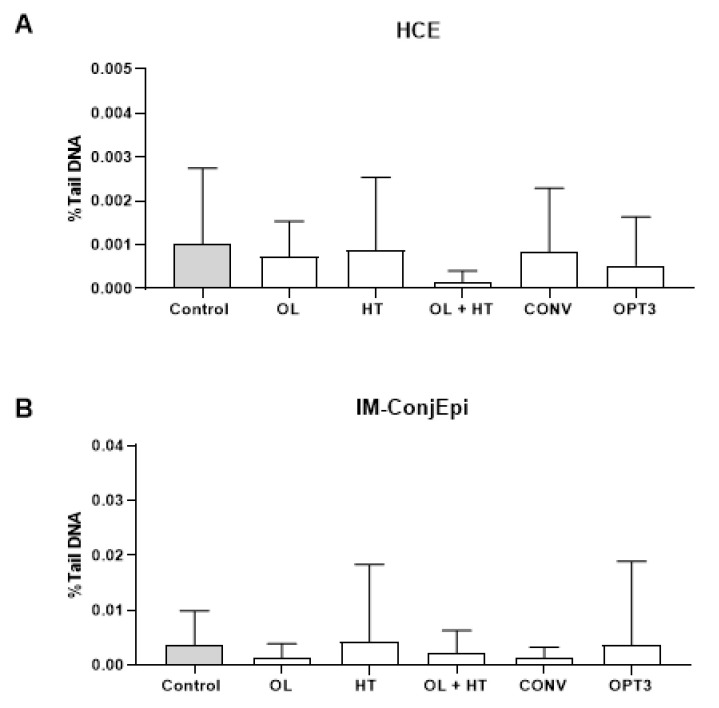
Genotoxic effect (alkaline comet assay) of aqueous solutions of olive pomace (OP) dried extracts (0.8 mg/mL conventional—CONV and 0.4 mg/mL optimized—OPT3), 300 μM (162.2 mg/L) oleuropein (OL), 100 μM (15.4 mg/L) hydroxytyrosol (HT) and their mixture (5 μM/2.7 mg/L + 50 μM/7.7 mg/L OL+HT) on human corneal (HCE) (**A**) and conjunctival (IM-ConjEpi) (**B**) epithelial cells treated for 24 h. No statistically significant differences have been observed between any of the treatment groups and the control group neither on HCE nor on IM-ConjEpi cells. Results are presented as mean of percentage of DNA present in the comet tail (%TDNA) ± standard deviation (SD). Control cells were treated with vehicle (culture medium).

Figure 2 presents the effect of all compounds and extracts on the DNA of HCE and IM-ConjEpi cells, expressed as a percentage of DNA present in the comet tail (%TDNA). From the ROUT analysis, no outliers due to biological diversity or technical errors were detected. From the ANOVA analysis (*p*-values included in Table S4 of the Supplementary Material), no statistically significant differences between any of the treatment groups and the control group (vehicle: culture medium) were found. Hence, no significant genotoxic effect was produced on HCE and IM-ConjEpi cells, either from CONV and OPT3 or from OL and HT (alone or in mixture) at the selected concentrations. Similarly, Di Mauro et al. [18] found that a purified fraction of olive mill wastewater did not show a genotoxic effect in statens seruminstitut rabbit cornea cells at low concentrations, although DNA damage was induced dose-dependently at higher concentrations.

Thus, all OP extracts and compounds tested in this work can be used safely used as topical ophthalmic products.

#### 2.4.4. Long-Term Storage Stability

##### Effect on % HT and OL Content

**Figure 3:**
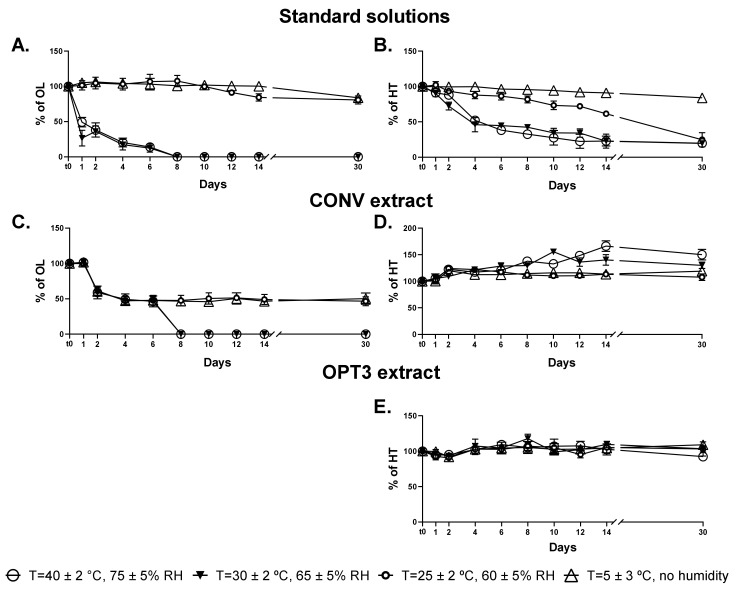
Stability studies up to 30 days of aqueous solutions of oleuropein (OL) standard (**A**), hydroxytyrosol (HT) standard (**B**), OL (**C**), and HT (**D**) in conventional (CONV) extract, and HT (**E**) in optimized (OPT3) extract at four different conditions of temperature (T) and relative humidity (RH). Results are presented as average of percentage of each compound (HT or OL) with respect to the initial quantity of t_0_ ± standard deviation (SD). Lines are added to guide the eye.

According to the guidance CPMP/ICH/2736/99, aqueous solutions of HT, OL, OPT3 extract, and CONV extract were stored at four different conditions of temperature (ambient, refrigerated, frozen, and intermediate) and humidity. The conditions were: T = 5 ± 3 °C (with no humidity), T = 25 ± 2 °C (with 60 ± 5% RH), T = 30 ± 2 °C (with 65 ± 5% RH) and T = 40 ± 2 °C (with 75 ± 5% RH) up to a final duration of 6 months. For OPT3, HT was relatively quantified as % with respect to the concentration (C_0_) at the initial time (t_0_), while for CONV both % HT and % OL content were calculated similarly. Figure 3 presents the variation during the first 30 days of storage for HT and OL, either as solutions of pure compounds (Figure 3A for OL and Figure 3B for HT) or as part of the extracts (Figure 3C for OL in CONV, Figure 3D for HT in CONV and Figure 3E for HT in OPT3). The stability of up to 6 months is presented in the Figure S1 of the Supplementary Material.

The experimental data of degradation obtained for HT and OL standard solutions during storage at the four different conditions were satisfactorily fitted to pseudo-first-order kinetics with lag phase (R^2^ > 0.8992) according to Equation (1) (t in days) [30,66]. The lag time (t_lag_) represents the time that the concentration of the compound remains constant and, if present, is determined as the intercept of the initial degradation time with the initial concentration value. The half-life period (t_1/2_) is calculated from Equation (2). The degradation constant (k_obs_), the t_1/2,_ and the time lag (t_lag_) together with the correlation coefficient (R^2^) for each compound and storage condition are presented in Table 5.

ln(C_0_/C_t_) = k_obs_(t − t_lag_) (1)

t_1/2_ = ln(2)/k + t_lag_ (2)

As a pure solution, OL is highly unstable at temperature values above 30 °C (Figure 3A), while remains at high levels at 5 °C or 25 °C (Figure 3A). From the kinetics results (Table 5), there is no t_lag_ for OL at a temperature above 30 °C, while the time to reduce the concentration to half of the initial value (t_1/2_) is around 3 days. However, for temperature values below 25 °C, OL remains stable up to approximately 11 days, having a high t_1/2_ in both 5 and 25 °C. HT as a standard solution starts to degrade almost from the first day but at a slower rate in comparison to OL (Figure 3B). Particularly, at 5 °C, HT is stable at a high percentage for the entire month. The degradation constant k_obs_ increased proportionally with temperature following an Arrhenius type equation (data not shown) for HT standard solution, as well as for OL, although in this case, the variation in concentration at 30 and 40 °C was nearly the same. Similarly, the t_1/2_ decreases proportionally from 101.5 days to 5.6 days as temperature increases for HT.

Nevertheless, HT and OL in the extracts demonstrate different degradation kinetics. This can be explained by the presence of more compounds of the same groups in the extract, which can be transformed/degraded to other molecules, such as HT [30,66]. For OL, the effect of the temperature above 30 °C is similar as part of the extract or pure solution (Figure 3C). However, regarding the temperatures of 5 °C and 25 °C, it can be observed that the stability of OL in CONV is different compared to the standard solution, with half of the initial quantity being degraded after 48 h (Figure 3C). On the other hand, HT concentration in the extracts remains stable or even increases. In particular, as part of OPT3 (Figure 3E), no degradation is observed during the first month, while it can be said that a slight increase occurs. For HT measured in CONV (Figure 3D), a significant increase in its content can be observed from day 2 which is even higher for temperatures above 30 °C. This could be explained by comparing the Figure 3C,D and taking into account the conversion of OL into HT [16,25,26]. After day 2, a decrease of the OL extract percentage is observed at all conditions, while for temperatures above 30 °C at day 8, OL is not detected. At the same time points, HT is increased at all conditions (day 2) and especially for high temperatures (day 8—T = 30 °C and 40 °C). These results support this hypothesis. For the OPT3, the presence of OLC in low quantities, a molecule that includes the “HT” moiety in its structure could also support the same premise. In the olive oil, Lozano-Sánchez et al. [66] has already proved the positive correlation between the increase of HT and the decrease of decarboxymethyl oleuropein aglycone, a molecule structurally similar to OLC. The low thermal stability of OL and HT has already been demonstrated [67,68]. However, it is important to highlight that the stability of these compounds in aqueous solutions is lower than in solid state [69] or in olive oil [30,66].

Regarding the stability of the compounds and extracts after the first month (Figure S1, Supplementary Material), OL standard solution (Figure S1A) at 5 °C and 25 °C is degraded proportionally with the time, reaching a final content of ca. 35% compared to the t_0_. In CONV (Figure S1C), OL is completely degraded at 25 °C from the second month and at 5 °C from the fifth month. HT as a standard solution (Figure S1B) at 30 °C and 40 °C is completely degraded from the third month and at 25 °C from the fourth month. However, at 5 °C, after 6 months, it remains at a concentration of approximately 60% compared to the initial. The increase of HT concentration in both extracts can be observed even more significantly after the first month, especially for CONV (Figure S1D). In fact, for temperatures above 30 °C, HT reaches a concentration of three to four times higher than that at t_0_. At temperatures below 25 °C, half of the initial quantity is degraded. Regarding OPT3 (Figure S1E), the tendency is similar to that of CONV extract. At temperatures above 30 °C, the final quantity of HT is 1.5–1.6 times higher, while at 25 °C or 5 °C, it remains at the same levels as at the end of the first month (ca. 110%). These long-term data support even stronger our hypotheses of molecules transformation within the extracts and their different stability kinetics as pure solutions or extracts.

From the results, it became mandatory to evaluate the chemical degradation profile of all solutions by HPLC-DAD-MS/MS.

##### HPLC-DAD-MS/MS Profile Comparison

To investigate the different stability profiles of HT and OL alone or as part of an extract, an HPLC-DAD-MS/MS analysis was performed for the pure HT, pure OL, CONV, and OPT3 after a 30, 6, 4 and 6 days exposure, respectively, at 40 °C. For CONV and OPT3, a total HPLC-DAD-MS/MS characterization using an electrospray ionization source in negative mode (ESI-) of the 40 °C exposed extracts was performed, putatively identifying all compounds present in them and comparing the phenolic profiles with those of the initial extracts (freshly prepared) (Table 4).

OL as a pure solution is degraded to two major compounds with *m/z* 137 and 403 (Figure S2, Supplementary Material) with 223 and 179 being the major fragments of *m/z* 403 confirming the existence of elenolic acid glucoside (compound 8—Table 4) [59]. This molecule is indeed part of the OL structure. On the other hand, the *m/z* 137 corresponds to the “4-ethylbenzene-1,2-diol” part of the OL molecule, left after the removal of the “elenolic acid glucoside” moiety. Thus, it can be said that the bond connecting the ester with the ethyl part is more susceptible to break when OL is a pure solution. However, when OL is part of the extract, its degradation profile seems different. In particular, the *m/z* 403 is also detected in the freshly prepared CONV extract (Table 4). Thus, elenolic acid glucoside can be a secondary metabolite present in the OP or be produced during the extraction by degradation/transformation of the molecules due to high temperature. In this case, it is not produced during the long-term storage of the extract solution. Additionally, an *m/z* 387 is detected in the extracts. This, together with the increase of HT (*m/z* 153) over time (Figure S1D) can confirm that OL, in this case, is degraded by breaking the bond inside the ester and producing the HT and Secologanin moieties.

Regarding HT aqueous pure solution, comparing the UV chromatogram at 280 nm with the ESI- scan chromatogram of the sample after 30 days exposure at 40 °C, the major mass detected had an *m/z* of 319 at 10.14 min (Figure S3, Supplementary Material). The same *m/z* was also detected at 13.28 min. Unfortunately, it was not possible to identify the compound corresponding to this mass, since all compounds existing in the bibliography with this *m/z* are not HT by-products. In addition, several peaks were detected in the sample: an *m/z* 303 at 26.38 min, an *m/z* 199 at 9.54 min, an *m/z* 151 at 8.73 min, an *m/z* 179 at 15.98 min, and an *m/z* 113 at 8.73 min, whose identification was not possible based on the existing bibliography. Further analyses with different identification techniques could help the identification of these degradation by-products in the future.

Attya et al. [67] used tandem mass spectrometry to evaluate the thermal stability of selected phenolic compounds of virgin olive oil. Regarding OL, a fragment with *m/z* 137 was formed during its thermal degradation, confirming our hypothesis, and demonstrating that in different materials/conditions, the degradation profile of the molecules may differ. However, in terms of HT, no specific fragments have been presented, due to a possible lack of clear spectrographic results. Some authors have proposed that HT is autoxidized to o-quinone or p-quinone methides that could form dimers possibly not detectable by HPLC-MS/MS after the addition of water [70,71].

## 3. Materials and Methods

### 3.1. Plant Material and Pretreatment Conditions

OP obtained from the processing of Arbequina variety (2018 crop) was kindly given by Oliduero (Medina del Campo, Spain). Detailed characterization of the initial raw material is presented in Table 6. Moisture was determined by drying the material at 105 °C until stable weight. Fat and extractives were defined by 3 consecutive extractions in Soxhlet using 3 different solvents: *n*-hexane for 6 h (fat), and EtOH and water for ca. 18 h each (extractives). Protein content was determined by the Kjeldahl method (with a conversion factor of 6.25). The ash content corresponded to the char formed at 550 °C. All yield compositions are expressed per gram of dry OP.

**Figure 4:**
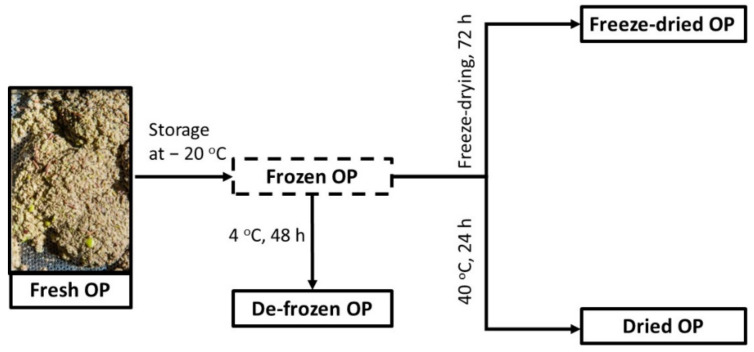
Four different storage conditions of the olive pomace (OP).

Four different pretreatment conditions have been tested for the raw material, as presented in Figure 4. Briefly, the fresh material was submitted to extraction upon arrival, without any previous pre-treatment. The rest of the material was then packed in plastic bags of approximately 1 kg with the use of N_2_ (to devoid oxygen) and stored at −20 °C for 4 months. The de-frozen OP was subjected to extraction directly after de-freezing, without any drying step. The freeze-dried OP was lyophilized under vacuum (18 kPa) and protected from light for 72 h. The dried OP was produced placing the frozen OP at a chamber of 40 °C for 24 h for slow drying. The moisture of the fresh and the de-frozen OP was 59.7 ± 0.2% (1.48 ± 0.01 g_H_2_O_/g_DRY OP_) (Table 6). The freeze-dried and the dried material had ca. 3% and 45% of moisture, respectively, and were also subjected to extraction directly after the pretreatment took place. Then, they were stored at room temperature, protected from light and moisture for up to 6 months.

### 3.2. Materials, Reagents and Solvents

Milli-Q water was obtained from a Millipore unit. EtOH non denaturalized (99.9%) was bought from Dávila Villalobos S.L. (Valladolid, Spain), N_2_ (99.996%) from Linde Gas (Puçol, Spain), Fluorescein sodium salt from Vetec Química (Xerem Duque De Caxias, Rio de Janeiro, Brazil) and OxiSelect 96-Well Comet Assay Kit from bioNova scientific (Fremont, CA, USA). Methanol (MeOH, 99.9% LC-MS), *n*-hexane (95%), NaOH pellets, Dimethyl Sulfoxide (DMSO), and phosphoric acid were supplied by Panreac Quimica SLU (Barcelona, Spain), while commercial standards HT (≥98%), TY (≥99%) and OL (≥98%) by Extrasynthese (Genay, France). MgSO_4_ anhydrous, KI, NaBr, NaCl, Trolox (6-hydroxy-2,5,7,8-tetramethylchromane-2-carboxylic acid), AAPH (2,2′-azobis(2-methylpropionamidine)dihydrochloride), gallic acid, catechin, Tris-EDTA buffer solution, and bovine insulin were purchased from Sigma-Aldrich (Madrid, Spain). Plastic culture flasks, plates, tips, and pipettes, Dulbecco’s modified Eagle’s medium/nutrient mixture F-12 (DMEM/F-12) + GlutaMax, Dulbecco’s phosphate-buffered saline, fetal bovine serum, human epithelial growth factor, human insulin, penicillin, and streptomycin were supplied by Thermo Fisher Scientific (Rockford, IL, USA). CO_2_ (99.95%) was obtained by Carburos Metálicos (Barcelona, Spain).

### 3.3. Phenolic Extraction

**Figure 5:**
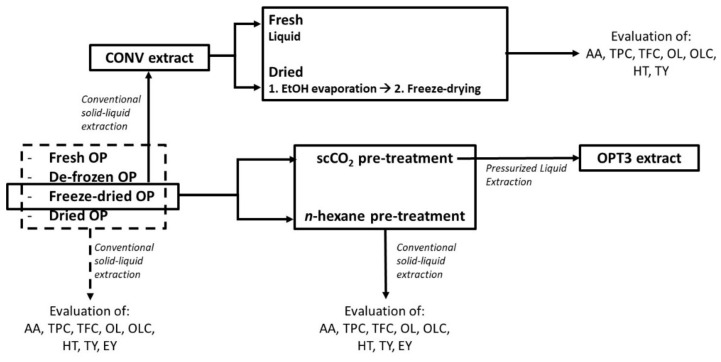
Experimental procedure followed for the phenolic extraction of the differently pretreated olive pomace (OP) and the effect of the extract drying. All extracts were evaluated in terms of antioxidant activity (AA) and richness in total phenolic content (TPC), total flavonoid content (TFC), oleuropein (OL), oleacein (OLC), hydroxytyrosol (HT), and tyrosol (TY). The extracts derived by the differently pretreated OP were also evaluated in terms of extraction yield (EY).

The experimental scheme regarding the phenolic extraction of the differently stored and pre-treated OP is presented in Figure 5. The experimental procedure for each step is described below in detail.

#### 3.3.1. Conventional Process: Effect of Different Pretreatment Conditions

To study the effect of the pretreatment conditions on the raw material, all materials (fresh, de-frozen, freeze-dried, or dried) were subjected to the same conventional solid–liquid phenolic extraction. The conditions were selected based on industrial constraints (principally solvent consumption) according to Álvarez [12] and Katsinas et al. [16], who also describe the extraction process in detail. Briefly, the CONV extract was produced using freeze-dried OP without any additional pre-treatment and applying a T of 70.0 °C, a %EtOH of 50.0%, and a solid/liquid ratio (S/L) of 0.5 g_RAW OP_/mL_SOLVENT_. The same extraction process and conditions were applied to all the differently pretreated OP. All extractions were performed in triplicate.

#### 3.3.2. Conventional Process: Defatting Pretreatment Step Selection

Two different defatting methods were applied to the freeze-dried OP: a conventional one using *n*-hexane and a scCO_2_ extraction. The conditions used for each process have been selected considering industrial constraints and are described by Katsinas et al. [16]. The percentage of the yield of the oil obtained was calculated gravimetrically by weighing the OP before and after the process. Both defatting processes were performed in triplicate. Following this, the two differently defatted OP were subjected to a conventional solid–liquid phenolic extraction as previously described. Phenolic extractions were also performed in triplicate.

#### 3.3.3. PLE Process

For the preparation of the OPT3 extract, the freeze-dried OP was defatted with scCO_2_ and then, a PLE extraction was performed, according to conditions previously optimized [16], to obtain an extract enriched in HT, TY, and TPC. Thus, a T of 184.0 °C, a %EtOH of 90.0%, and a S/L of 0.8 g_RAW OP_/mL_SOLVENT_ were used.

All phenolic extracts remained in liquid form after production and were stored in darkness and at −20 °C until analysis.

### 3.4. Extract Drying

The effect of the drying process on the produced extract has been studied using the CONV extract (Figure 5). After the extraction, part of CONV was kept in liquid form (reference) and the rest was dried in two sequential steps. Briefly, CONV was transferred to a round bottom flask and, using a rotary evaporator (Buchi Rotavapor R-200, Flawil, Switzerland), the EtOH was evaporated at 60 °C and ca. 20 kPa (step 1) for approximately 20 min. The extract containing principally water was lyophilized under vacuum (18 kPa) and in darkness for 72 h (Lyoquest-55, Telstar, Terrassa, Spain) (step 2). The liquid sample after step 1 was filtered using a 0.20 μm polyvinylidene fluoride filter to remove any residual soil. The final dried extract (after step 2) was reconstituted in EtOH:H_2_O = 50:50 at 5.0 mg/mL and filtered similarly. Both samples were characterized together with the freshly prepared liquid extract (reference). From the already determined EY of the CONV extract, all results were calculated and expressed as mg of compound/compound equivalents or mmol of TE (for AA) per g of DE. The procedure was performed in duplicate.

### 3.5. Extract Characterization: Pretreatment Effect, Defatting Process and Extract Drying

The extracts produced by the differently pretreated or defatted OP were characterized in terms of EY, TPC, TFC, AA (determined by the ORAC assay), and richness in major phenolic compounds (HT, OL, TY, and OLC) (Figure 5). The differently dried extracts were reconstituted (ca. 5.0 mg/mL) and were characterized similarly, except the EY. The procedures followed for all the aforementioned characterizations are described in detail by Katsinas et al. [16] The richness in HT, OL, TY, and OLC was determined by HPLC-DAD as described below. The results for EY are expressed as mg of DE/g of dry OP, for TPC as mg of gallic acid equivalents (GAE)/g DE, for TFC as mg of catechin equivalents (CATE)/g DE, and for AA as mmol of Trolox equivalents (TE)/g DE. The extract richness in the selected phenolic compounds is expressed as mg of compounds/g DE. OLC was calculated as OL equivalents (OLE).

### 3.6. HPLC-DAD Analysis

The quantitative determination of HT, TY, and OL was performed by an HPLC-DAD system: Waters e2695 separation module with an autosampler (20 μL injection volume) and a quaternary pump, coupled with Waters 2998 photodiode array detector set at 280 nm (Waters^®^, Dublin, Ireland). A C18 Mediterranean Sea column (250 × 4.6 mm, 5 μm) (Teknokroma Analítica S.A., Barcelona, Spain) at 35 °C connected with an OptiGuard 1 mm guard column (Sigma-Aldrich, St. Louis, MO, USA) were used. The gradient method, together with the eluents and the elution program used, were selected according to Katsinas et al. [16]. Calibration curves for OL (range: 12.5–1250 mg/L, linearity: R^2^ = 0.9989), HT (range: 25–300 mg/L, linearity: R^2^ = 0.9992), and TY (range: 12.2–200 mg/L, linearity: R^2^ = 0.9969) were prepared, using standard solutions of the compounds in DMSO, and analyzed in the same conditions as the samples. Compounds in the extracts were identified by comparing the retention time and the UV spectra of the samples with those of the standard solutions, while MS spectra were also used to confirm identification (see 3.7.1. for experimental conditions). The accuracy was determined as the level of agreement between the results of analysis of 3 independent analyte samples of known concentration and the true value. It was expressed as % relative error ± standard deviation (SD) among the independent samples and calculated as −1.6 ± 0.8% for OL, −4.7 ± 3.8% for HT, and −3.3 ± 2.8% for TY. The precision/repeatability was determined as the degree of agreement among 3 individual test results of the same analyte sample and was calculated as 3.2% relative SD for OL, 2.2% for HT, and 6.8% for TY. Samples and calibration curves of standard solutions were analyzed within the same day. For the 6-months storage stability, relative quantification was performed with respect to the initial quantity t_0_ of each analyte. For data acquisition and processing, Empower^®^ 3 software (Waters^®^, Dublin, Ireland) was used.

### 3.7. Composition, Stability and Genotoxicity Characterization towards Ophthalmic Applications of Aqueous Solutions of Selected Extracts (CONV, OPT3) and Pure Compounds (HT and OL)

#### 3.7.1. HPLC-DAD-MS/MS Analysis

The phenolic compounds present in the freshly prepared aqueous solutions of CONV and OPT3, and the degradation phenolic products present in the 40 °C exposed aqueous solutions of OL, HT, CONV, and OPT3, were putatively identified by an HPLC-DAD-MS/MS system: Waters Alliance 2695 (Waters^®^, Dublin, Dublin, Ireland) separation module with an autosampler (10 μL injection volume), a quaternary pump and a solvent degasser, coupled to a Photodiode Array Detector Waters 996 PDA (Waters^®^, Dublin, Ireland) scanning wavelength absorption between 210 and 600 nm. A LiChrospher^®^ 100 RP-18 5 μm (250 × 4.0 mm) (Sigma-Aldrich, St. Louis, MO, USA) column at 35 °C (stabilized by a column oven) was used. MS/MS detection was carried out with a Micromass^®^ Quattro Micro triple quadrupole (Waters^®^, Dublin, Ireland), using an ESI. A full scan mode (*m/z*: 60–1100) record was applied for the mass spectra of the compounds separated by HPLC, using a collision energy of 20 eV. The HPLC gradient method, eluents, and elution program used, together with the source temperature, capillary, and source voltages are described by Katsinas et al. [16] For data acquisition and processing, MassLynx^®^ 4.1 software (Waters^®^, Dublin, Ireland) was used.

#### 3.7.2. In vitro genotoxicity Assay

##### Cell Culture

Two different ocular surface epithelial cell lines were used for the genotoxicity assays: the HCE and the IM-ConjEpi.

HCE is an immortalized human corneal epithelial cell line [72], kindly offered by Dr. Arto Urti (University of Helsinki, Finland). It was cultured in DMEM/F-12 + GlutaMax supplemented with 10% fetal bovine serum, 10 ng/mL human epithelial growth factor, 5 μg/mL human insulin and antibiotics (100 U/mL penicillin + 0.1 mg/mL streptomycin). IM-ConjEpi was supplied by Innoprot (Derio, Spain. Ref. P10870-IM) and is an SV40- Large T Antigen immortalized human conjunctival epithelial cell line. It was cultured in DMEM/F-12 + GlutaMax supplemented with 10% fetal bovine serum, 10 ng/mL human epithelial growth factor, 1 μg/mL bovine insulin and antibiotics (5000 U/mL penicillin + 5000 μg/mL streptomycin). HCE cell line was used from passage 25 to 35, while IM-ConjEpi from passage 10 to 20.

The cells were incubated at 37 °C and 5:95 = CO_2_:air atmosphere ratio, while their medium was changed every second day. Observations through a phase-contrast microscope were carried out daily.

##### Preparation of Treatment Phenolic Solutions

For the in vitro tests, HT, OL, CONV, and OPT3 were dissolved in culture medium (DMEM/F-12 + GlutaMax, without any supplement) the day of the experiment. As previously described, CONV was produced by conventional solid–liquid extraction using freeze-dried material without any further pretreatment. OPT3 was generated by PLE extraction using freeze-dried material previously defatted with scCO_2_. The part of the extracts not dissolved to the culture medium was filtered through a 0.20 μm polyvinylidene fluoride sterile filter. The in vitro concentrations were selected according to a previous study, which described the maximum allowable non-toxic concentration of HT, OL, CONV, and OPT3 on the two ocular surface cell lines used [17]. Thus, HT was tested at a concentration of 100 μM (equivalent to 15.4 mg/L), OL at 300 μM (equivalent to 162.2 mg/L), CONV at 80 mg/L and OPT3 at 40 mg/L. A mixture of 5 μM of OL (2.7 mg/L) with 50 μM of HT (7.7 mg/L) was prepared by mixing the double concentration of each compound in equal volumes.

##### Comet Assay

To test the in vitro genotoxicity effect of the phenolic solutions, the comet assay was selected. The protocol was performed on HCE and IM-ConjEpi cells as previously described by Di Mauro et al. [73], with some modifications. According to the manufacturer’s instructions, the cells mixed with liquid agarose were placed on an agarose-precoated 96-well slide and left at 4 °C for 15 min until solidification. Then, the slide was immersed in lysis buffer (2.5 M NaCl, 100 mM Na2EDTA, 10 mM Tris–HCl, 1% n-laurosil-sarcosine, 1% Triton X-100, 10% DMSO, pH 10) at 4 °C for 60 min. Subsequently, it was transferred to a container filled with pre-chilled alkaline solution (300 mM NaOH, 1 mM Na2EDTA) and left immersed at 4 °C for 30 min to allow DNA to unroll. Following this, using the same alkaline solution, alkaline electrophoresis was performed for 30 min at 1 volt/cm and 300 mA. Finally, the slide was washed 3 times with pre-chilled distilled H_2_O, dried with 70% ethanol, and stained with vista green DNA dye for 15 min in darkness. Images were acquired with an inverted epi-fluorescence microscope (DMI 6000 B, Leica, Wetzlar, Germany) using a fluorescein isothiocyanate filter. The images were analyzed using the CASP (1.2.3) image analysis software. Three independent experiments were performed, analyzing ca. 50 cells/treatment for each experiment. Results are expressed as mean of %TDNA ± SD.

##### 3.7.3. Long-Term Storage Stability

**Figure 6:**
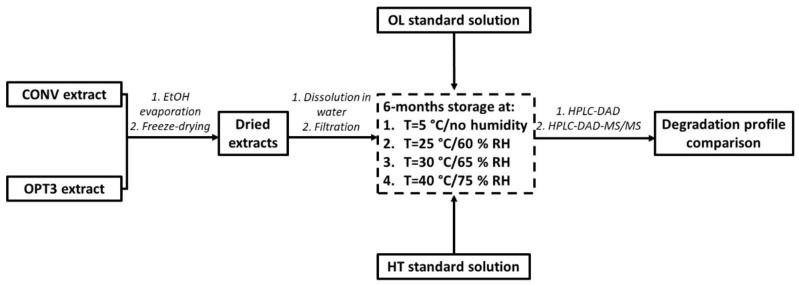
Experimental procedure of the stability studies performed for aqueous solutions of oleuropein (OL) and hydroxytyrosol (HT) standards, as well as of conventional (CONV) and optimized (OPT3) extracts at 4 different conditions of temperature (T) and relative humidity (RH).

The experimental procedure of the relative stability studies is presented in Figure 6. Briefly, all compounds and extracts were dissolved in Milli-Q water, as high mineralization could increase the degradation of these substances [68]. Additionally, according to the bibliography [68], lower concentrations could accelerate the decomposition of a compound. Hence, higher concentrations have been selected to be measured for all compounds and extracts. CONV and OPT3 were prepared at a concentration of 5.0 mg/mL, HT at 72 mg/L, and OL at 113 mg/L. All solutions were filtered through a 0.20 μm polyvinylidene fluoride filter. A total of 3 mL of each phenolic aqueous solution was added to amber glass vials, degassed with N_2,_ closed airtight with a rubber stopper, and sealed with aluminum capsules. To fix the desired humidity for each temperature, appropriate salts have been used according to the bibliography [74]. For 5 °C, the MgSO_4_ anhydrous was selected (to achieve no humidity), for 25 °C: NaBr (to reach a 57.57% ± 0.40 RH), for 30 °C: KI (setting the RH at 67.89% ± 0.3) and for 40 °C: NaCl (establishing an RH of 74.68% ± 0.13). The vials were placed in big glass containers, together with a sufficient quantity of each salt. Then, they were closed airtight and left in different rooms of controlled temperature and protected from light for 6 months. Samples were taken immediately after preparation (considered as t_0_) and afterward at days 1 (24 h), 2, 4, 6, 8, 10, 12, 14, and 30 (1 month). After the first month, one sample was taken every 30 days, up to a total duration of 6 months. For CONV, HT and OL content were measured, while for OPT3 only HT content was calculated due to the lack of OL in this extract. All samples were analyzed in triplicate and the results are expressed as mean of the percentage with respect to the initial concentration t_0_ ± SD.

### 3.8. Statistical Analysis

Levene’s test was used to examine the homogeneity of variances, while one-way ANOVA with Tukey’s or Games–Howell post hoc test was performed for intergroup comparisons. For the in vitro results, ROUT analysis was used to identify possible outliers. *p*-values lower than 0.05 were determined as statistically significant. For the statistical analysis, the SPSS software (SPSS 15.0; SPSS, Inc., Chicago, IL, USA) was used, while for kinetics data treatment the Microsoft Excel software (MS Excel 10.0; Microsoft, Redmond, WA, USA) was used.

## 4. Conclusions

The results of this study demonstrate how a usually discarded and potentially environmentally hazardous agro-industrial by-product, the OP, can be transformed into a future ophthalmic product, which is usually aqueous-based. Different pretreatment conditions were evaluated for the raw material in terms of their effect on the richness of its major phenolic compounds in the hydroalcoholic extracts produced. The lyophilization of the raw material and the subsequent scCO_2_ extraction of the residual oil were proved to be the most suitable. Subsequently, following the EMA industrial guidelines for a drug product, the ophthalmic safety and the 6-month storage stability of aqueous solutions of pure OL and HT, and two selected OP extracts were evaluated. The selection of the OP extracts (CONV: produced by conventional extraction using freeze-dried material, and OPT3: produced by PLE using scCO_2_-defatted freeze-dried OP) was based on previous in vitro ocular anti-inflammatory and antioxidant activity results and irritation studies. Regarding safety, the genotoxic effect (comet assay) was studied on two different human ocular surface epithelial cell lines, proving the use of all compounds and extracts to be safe at the concentrations tested. Regarding long-term storage stability, OL and HT demonstrated a remarkably different stability profile as pure or extract solutions at the four different temperature/moisture conditions tested. OL in CONV was highly degraded (ca. 50%) after day 2 in all conditions, while on the contrary as a standard solution was stable for up to 1 month at 25 °C or below. HT as a pure solution demonstrated good stability only at 5 °C. However, as part of OPT3, HT remained stable or was even increased over time proportionally with the reduction of secoiridoids at all conditions. The same tendency was observed for HT in CONV above 30 °C. An HPLC-DAD-MS/MS analysis of the degraded solutions allowed us to identify the different degradation profiles of OL alone or in the extract. The interactions between OL and HT, and the degradation profile of OL aqueous solutions were established for the first time. Hence, a necessary evaluation baseline for the future approval of OP extracts and their major compounds as ophthalmic products was proposed. Future formulation studies are necessary to increase the stability of the composition in the extract or compounds for its final application.

## Figures and Tables

**Table 1:** Results obtained for extracts generated by conventional phenolic extraction conditions, using olive pomace (OP) pretreated at different conditions. Values with different lowercase letters in the same column are significantly different (*p* < 0.05).

Material	AA (mmol_TE_/g_DE_)	TPC (mg_GAE_/g_DE_)	TFC (mg_CATE_/g_DE_)	OL (mg/g_DE_)	OLC (mg_OLE_/g_DE_)	HT (mg/g_DE_)	TY (mg/g_DE_)	EY (mg_DE_/g_DRY OP_)
**Fresh**	3.64 ± 0.15 ^bc^	117 ± 11 ^a^	8.0 ± 0.7 ^b^	6.0 ± 0.8 ^b^	3.7 ± 0.7 ^b^	1.0 ± 0.3 ^bc^	1.5 ± 0.3 ^ab^	134 ± 15 ^b^
**De-frozen**	3.8 ± 0.3 ^b^	130 ± 1 ^a^	10.9 ± 0.4 ^a^	10.1 ± 1.3 ^c^	5.2 ± 0.3 ^b^	1.4 ± 0.5 ^ac^	1.3 ± 0.2 ^b^	116 ± 4 ^ab^
**Freeze-dried**	4.36 ± 0.08 ^a^	131 ± 27 ^a^	11.2 ± 1.3 ^a^	3.4 ± 0.5 ^a^	12.0 ± 3.3 ^a^	1.9 ± 0.3 ^a^	1.9 ± 0.2 ^a^	94 ± 6 ^a^
**Dried**	3.2 ± 0.12 ^c^	105.0 ± 0.5 ^a^	5.0 ± 0.2 ^c^	2.53 ± 0.01 ^a^	2.4 ± 0.3 ^b^	0.17 ± 0.07 ^b^	0.28 ± 0.01 ^c^	112 ± 6 ^ab^

Results are presented as average ± standard deviation (SD). Responses measured: antioxidant activity (AA—expressed as mmol of Trolox equivalents (TE)/g dry extract (DE)); total phenolic content (TPC—expressed as mg of gallic acid equivalents (GAE)/g DE; total flavonoid content (TFC—expressed as mg of catechin equivalents (CATE)/g DE), extract richness in oleuropein (OL), oleacein (OLC), hydroxytyrosol (HT), and tyrosol (TY) (expressed as mg of compound/g DE, OLC was calculated as OL equivalents: OLE); and extraction yield (EY—expressed as mg of DE/g dry OP).

**Table 2:** Effect of the two defatting pretreatment methods selected on the responses measured for conventional phenolic extracts produced using freeze-dried olive pomace (OP). Values with different lowercase letters in the same column are significantly different (*p* < 0.05).

Material	AA (mmol_TE_/g_DE_)	TPC (mg_GAE_/g_DE_)	TFC (mg_CATE_/g_DE_)	OL (mg/g_DE_)	OLC (mg_OLE_/g_DE_)	HT (mg/g_DE_)	TY (mg/g_DE_)	EY (mg_DE_/g_DRY OP_)
**Non-defatted freeze-dried OP** (**Reference**)	4.36 ± 0.08 ^a^	131 ± 27 ^a^	11.2 ± 1.3 ^a^	3.4 ± 0.5 ^a^	12.0 ± 3.3 ^a^	1.9 ± 0.3 ^a^	1.9 ± 0.2 ^a^	94 ± 6 ^a^
**Freeze-dried OP defatted with *n*-hexane**	4.8 ± 0.5 ^a^	152 ± 15 ^a^	9 ± 3 ^a^	2.6 ± 0.2 ^a^	12.1 ± 1.3 ^a^	1.9 ± 0.2 ^a^	1.8 ± 0.2 ^a^	93 ± 11 ^a^
**Freeze-dried OP defatted with Supercritical CO_2_**	4.66 ± 0.14 ^a^	180 ± 11 ^b^	11.2 ± 1.3 ^a^	3.3 ± 0.8 ^a^	11.8 ± 1.6 ^a^	1.80 ± 0.1 ^a^	1.78 ± 0.10 ^a^	121 ± 25 ^a^

Results are presented as average ± Standard deviation (SD) and compared with the non-defatted OP. Responses measured: Antioxidant activity (AA—expressed as mmol of Trolox equivalents (TE)/g dry extract (DE)), Total phenolic content (TPC—expressed as mg of gallic acid equivalents (GAE)/g DE, Total flavonoid content (TFC—expressed as mg of catechin equivalents (CATE)/g DE), extract richness in oleuropein (OL), oleacein (OLC), hydroxytyrosol (HT) and tyrosol (TY) (expressed as mg of compound/g DE, OLC was calculated as OL equivalents: OLE), as well as extraction yield (EY—expressed as mg of DE/g dry OP).

**Table 3:** Effect of extract drying on the retention of the responses measured, compared to a freshly obtained liquid extract (reference: conventional phenolic extract produced using freeze-dried olive pomace (OP)). Step 1 included only ethanol (EtOH) evaporation (extract in liquid state), followed by step 2: freeze-drying of the extract (extract in solid-state). Values with different lowercase letters in the same column are significantly different (*p* < 0.05).

		AA (mmol_TE_/g_DE_)	TPC (mg_GAE_/g_DE_)	TFC (mg_CATE_/g_DE_)	OL (mg/g_DE_)	OLC (mg_OLE_/g_DE_)	HT (mg/g_DE_)	TY (mg/g_DE_)
**Reference: Fresh-Liquid Extract**		4.36 ± 0.08 ^a^	131 ± 27 ^a^	11.2 ± 1.3 ^a^	3.4 ± 0.5 ^a^	12.0 ± 3.3 ^a^	1.9 ± 0.3 ^a^	1.9 ± 0.2 ^a^
**Drying process**	**Step 1**	4.24 ± 0.15 ^a^	128 ± 15 ^a^	11.3 ± 0.9 ^a^	3.2 ± 0.3 ^a^	11.5 ± 2.4 ^a^	1.65 ± 0.05 ^a^	1.91 ± 0.10 ^a^
	**Step 2**	2.4 ± 0.2 ^b^	70 ± 10 ^b^	10.4 ± 1.0 ^a^	5.4 ± 0.8 ^b^	7.8 ± 1.2 ^a^	1.00 ± 0.12 ^b^	1.1 ± 0.3 ^b^

Results are presented as average ± Standard deviation (SD). Responses measured: antioxidant activity (AA—expressed as mmol of Trolox equivalents (TE)/g dry extract (DE)); total phenolic content (TPC—expressed as mg of gallic acid equivalents (GAE)/g DE; total flavonoid content (TFC—expressed as mg of catechin equivalents (CATE)/g DE) and extract richness in oleuropein (OL), oleacein (OLC), hydroxytyrosol (HT), and tyrosol (TY) (expressed as mg of compound/g DE, OLC was calculated as OL equivalents: OLE).

**Table 4:** Putative identification of phenolic compounds in the aqueous solution of conventional (CONV) and optimized (OPT3) dried extracts. The table includes the peak numbers as mentioned in the chromatogram (Figure 1A,B), together with the retention time, molecular formula, [M-H]- ion, major electrospray ionization source in negative mode (ESI−) product ions for each compound, and the sample in which they appear.

Peak nº	Putative Identification	Chemical Class	Molecular Formula	Retention Time (min)	Precursor Ion [M-H]^−^ (*m/z*)	Product Ion (*m/z*)	References	Presence in Sample
1	Quinic acid	Hydroxybenzoic acid	C_7_H_11_O_6_	8.22	191	173, 133, 127, 111, 85	[59]	CONV, OPT3
2	Hydroxytyrosol glucoside	Glucoside	C_14_H_20_O_8_	10.22	315	153, 135, 123, 89	[60]	CONV, OPT3
3	Unknown compound 1	-	-	10.84	407	289, 176, 151, 124, 89		CONV
4	Hydroxytyrosol (HT)	Simple phenol	C_8_H_10_O_3_	12.25	153	123	[60]	CONV, OPT3
18	Unknown compound 3	-	-	12.84	143	161 (water adduct of 143), 99, 71, 45		OPT3
5	Dialdehydic elenolic acid decarboxymethyl (DEDA)	Secoiridoid	C_9_H_12_O_4_	13.61	183	139, 95, 69	[16]	CONV
19	Unknown compound 4	-	-	13.84	219	111, 87, 67		OPT3
6	Tyrosol (TY)	Simple phenol	C_8_H_10_O_2_	16.78	137	134, 119, 108, 84, 47	[59]	CONV, OPT3
20	Vanillin	Aldehyde	C_8_H_7_O_3_	18.76	151	123, 108	[59,61]	OPT3
7	Secologanoside/Oleoside	Secoiridoid glycosides	C_16_H_22_O_11_	18.94	389	165, 121, 119, 89, 69	[16,59]	CONV
8	Elenolic acid glucoside	Secoiridoid	C_17_H_23_O_11_	19.35	403	223, 179, 119, 101, 89, 59	[59]	CONV
9	Unknown compound 2	-	-	20.61	671	335, 151		CONV
21	7-epiloganin	Iridoid	C_16_H_22_O_11_	23.83	389	151, 101, 89	[59]	OPT3
10	Oleuropein aglycone derivative	Secoiridoid	C_19_H_22_O_8_	25.27	377	217, 197, 153, 84	[16]	CONV
22	Elenolic acid hexoside derivative	Secoiridoid	C_20_H_34_O_13_	29.01	481	371, 165, 151	[61]	OPT3
11	Hydroxyoleuropein	Secoiridoid	C_25_H_32_O_14_	30.60	555	455, 323, 223, 151	[59]	CONV
23	Unknown compound 5	-	-	33.85	247	139, 111, 87, 41		OPT3
24	Hydroxytyrosol acetate	Secoiridoid	C_10_H_11_O_4_	35.63	195	135, 59	[59]	OPT3
12	Verbascoside	Secoiridoid glycoside	C_29_H_36_O_15_	37.78	623	461, 161	[16]	CONV
13	Elenolic acid derivative	Secoiridoid	C_11_H_14_O_6_	41.67	241	139, 127, 111, 101, 95, 69	[16]	CONV
14	Nüzhenide	Secoiridoid	C_31_H_42_O_17_	45.92	685	523, 453, 432, 421, 348, 299, 223, 119	[16]	CONV
25	Unknown compound 6	-	-	46.36	239	150, 80, 59		OPT3
15	Oleacein	Secoiridoid	C_17_H_20_O_6_	49.32	319	195, 139, 95, 69	[16]	CONV, OPT3
16	Oleuropein (OL)	Secoiridoid glycoside	C_25_H_32_O_13_	58.20	539	441, 377, 341, 307, 275, 223, 179, 149, 119, 89	[16,59,61]	CONV

**Table 5:** Hydroxytyrosol (HT) and Oleuropein (OL) pure aqueous solutions first-order degradation rate constants (k_obs_), half-time period (t_1/2_), and lag time (t_lag_) during accelerated stability studies at four different temperature (T)/relative humidity (RH) conditions.

Sample	Storage Conditions	k_obs_ (Days^−1^)	t_lag_ (Days)	t_1/2_ (Days)	R^2^ (-)
HT	T = 5 °C/no humidity	0.0069	1.0	101.5	0.9796
	T = 25 °C/60% RH	0.0337	1.0	21.6	0.9846
	T = 30 °C/65% RH	0.1068	0	6.5	0.9691
	T = 40 °C/75% RH	0.1234	0	5.6	0.9800
OL	T = 5 °C/no humidity	0.0099	11.3	81.3	0.9833
	T = 25 °C/60% RH	0.0065	9.9	116.5	0.9732
	T = 30 °C/65% RH	0.4022	0	1.7	0.8992
	T = 40 °C/75% RH	0.3658	0	1.9	0.9694

**Table 6:** Olive pomace (OP) characterization.

Moisture	Fat	Ash	Protein	Extractives
g_H2O_/g_DRY OP_	mg/g_DRY OP_	mg/g_DRY OP_	mg/g_DRY OP_	mg/g_DRY OP_
1.48 ± 0.01	200 ± 6	25.2 ± 1.7	143 ± 4	479 ± 7

## Data Availability

Not applicable.

## Supplementary Materials

The following are available online. Figure S1: Stability studies from 1 to 6 months of OL standard, HT standard, OL and HT in CONV, and HT in OPT3 at 4 different conditions of T and RH, Figure S2: Full scan mass spectra acquired in ESI- from peaks at retention time 15.91 min and 18.83 min detected in the analysis of OL aqueous standard solution after exposure for 2 days at 40 ± 2 °C and 75 ± 5% RH, and scan chromatogram, Figure S3: UV Chromatogram at 280 nm, scan chromatogram in ESI− and full scan mass spectra from peak at retention time 10.14 min detected in the analysis of HT aqueous solution after 30 days exposure at 40 ± 2 °C and 75 ± 5% RH, Table S1: ANOVA of all responses measured for extracts generated by conventional phenolic extraction conditions, using OP stored at different conditions, Table S2: ANOVA of the effect of 2 different defatting methods on the responses measured for extracts generated by conventional phenolic extraction conditions, using freeze-dried OP, Table S3: ANOVA of the effect of the extract drying on the stability of the responses measured compared to a freshly obtained liquid extract (reference), Table S4: ANOVA of the genotoxic effect (alkaline comet assay) of OP extracts (0.8 mg/mL CONV and 0.4 mg/mL OPT3), together with 300 μM (162.2 mg/L) OL, 100 μM (15.4 mg/L) HT and their mixture (5 μM/2.7 mg/L + 50 μM/7.7 mg/L OL+HT) on HCE and IM-ConjEpi cells treated for 24 h, compared to control cells (treated with culture medium).

## Sample Availability

Not available.

## Abbreviations

%EtOH	Percentage (%) of ethanol in water
%TDNA	Percentage (%) of DNA present in the comet tail
AA	Antioxidant Activity
AAPH	2,2′-azobis(2-methylpropionamidine)dihydrochloride
ANOVA	Analysis of Variances
CATE	Catechin Equivalents
CONV	Conventional olive pomace extract
DE	Dry Extract
DMEM/F-12	Dulbecco’s Modified Eagle’s Medium/Nutrient Mixture F-12
DMSO	Dimethyl Sulfoxide
EMA	European Medicines Agency
ESI-	Electrospray Ionization Source in negative ion mode
EtOH	Ethanol
EY	Extraction Yield
GAE	Gallic Acid Equivalents
HCE	Human Corneal Epithelial cells
HT	Hydroxytyrosol
IM-ConjEpi	Immortalized Human Conjunctival Epithelial cells
k_obs_	Degradation constant
MeOH	Methanol
OL	Oleuropein
OL+HT	Combination of Oleuropein and Hydroxytyrosol
OLC	Oleacein
OLE	Oleuropein Equivalents
OP	Olive Pomace
OPT3	Optimized olive pomace extract
ORAC	Oxygen Radical Absorbance Capacity
PLE	Pressurized Liquid Extraction
RH	Relative Humidity
S/L	Solid/Liquid ratio
scCO_2_	supercritical carbon CO_2_
SD	Standard Deviation
T	Temperature
t_1/2_	Half-life period
TE	Trolox Equivalents
TFC	Total Flavonoid Content
t_lag_	Lag time
TPC	Total Phenolic Content
Trolox	6-hydroxy-2,5,7,8-tetramethylchromane-2-carboxylic acid
TY	Tyrosol
