# Supplementary material for: Olive Pomace Phenolic Compounds Stability and Safety Evaluation: From Raw Material to Future Ophthalmic Applications
Source: Molecules. 2021 Oct 2;26(19):6002. doi: 10.3390/molecules26196002 (PMC8512844; doi:10.3390/molecules26196002)
Supplement: Supplementary file 1 [file molecules-26-06002-s001.zip › molecules-1358858-supplementary.pdf]

Supporting information

Olive pomace phenolic compounds stability and safety evaluation: from raw material to future ophthalmic applications

Nikolaos Katsinas [1,2,3], Amalia Enríquez-de-Salamanca [2,3], Andreia Bento da Silva [4,5,6], Maria Rosário Bronze [4,7,8] and Soraya Rodríguez-Rojo [1,*]

[1] Research Institute on Bioeconomy (BioEcoUVa), High Pressure Processes Group, School of Engineering, University of Valladolid (UVa), Dr. Mergelina str., 47011, Valladolid, Spain; nkatsinas@ioba.med.uva.es
[2] Institute of Applied Ophthalmobiology (IOBA), Campus Miguel Delibes, University of Valladolid (UVa), Paseo de Belén 17, 47011 Valladolid, Spain; amalia@ioba.med.uva.es
[3] Biomedical Research Networking Center in Bioengineering, Biomaterials and Nanomedicine (CIBER-BBN), Av. Monforte de Lemos, 3-5, 28029 Madrid, Spain
[4] Instituto de Investigação do Medicamento (iMed.ULisboa), Faculdade de Farmácia, Universidade de Lisboa (FFULisboa), Av. Prof. Gama Pinto, 1649-019, Lisbon, Portugal; mrbronze@ff.ulisboa.pt; abentosilva@ff.ulisboa.pt
[5] Departamento de Ciências Farmacêuticas e do Medicamento (DCFM), Faculdade de Farmácia da Universidade de Lisboa (FFUL), Av. das Forças Armadas, 1649-003 Lisboa, Portugal
[6] Faculdade de Ciências e Tecnologia da Universidade Nova de Lisboa (FCT NOVA), Largo da Torre, 2829-516, Caparica, Portugal
[7] Instituto de Biologia Experimental e Tecnológica (iBET), Apartado 12, 2780-901, Oeiras, Portugal
[8] Instituto de Tecnologia Química e Biológica da Universidade Nova de Lisboa (ITQB NOVA), Av. da República, 2780-157, Oeiras, Portugal
* Correspondence: soraya.rodriguez@uva.es; Tel.: +34-983-184-077

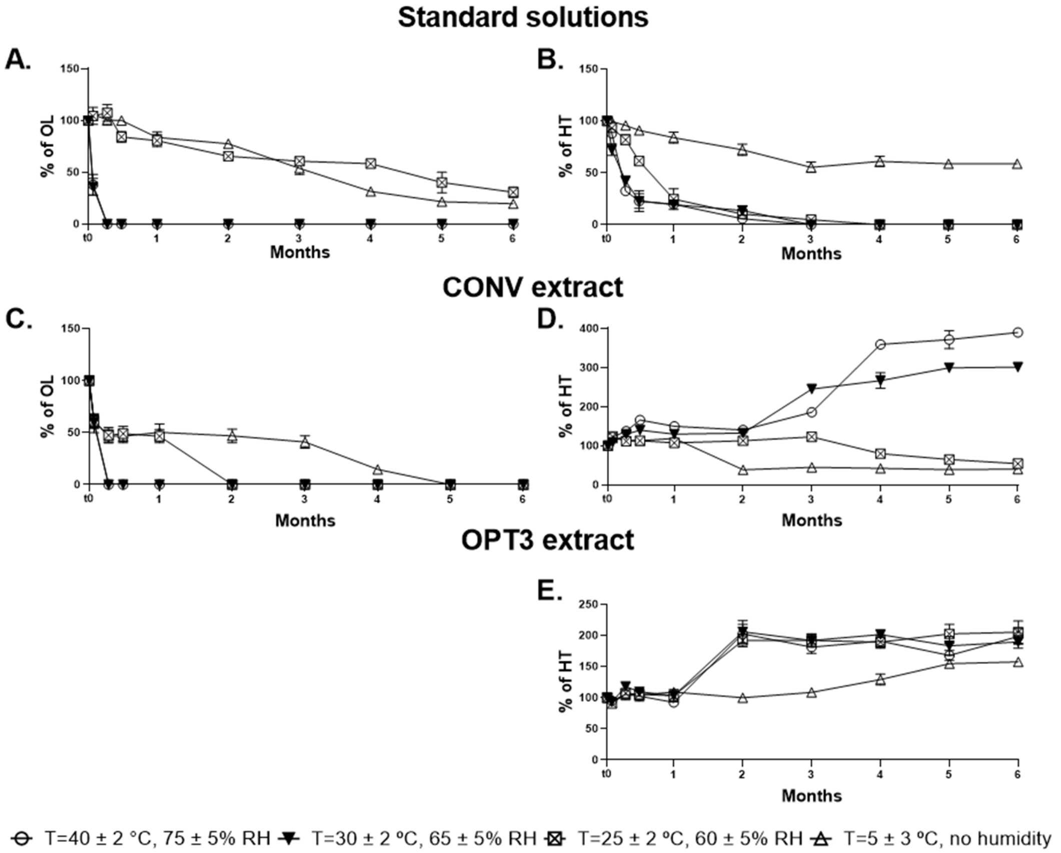

Figure S1. Stability studies from 1 to 6 months of aqueous solution of oleuropein (OL) standard (**A**), hydroxytyrosol (HT) standard (**B**), OL (**C**) and HT (**D**) in conventional (CONV) extract, and HT (**E**) in optimized (OPT3) extract at 4 different conditions of temperature (T) and relative humidity (RH). Results are presented as average of percentage of each compound (HT or OL) with respect to the initial quantity of t_0 ± standard deviation (SD). Lines added to guide the eye.

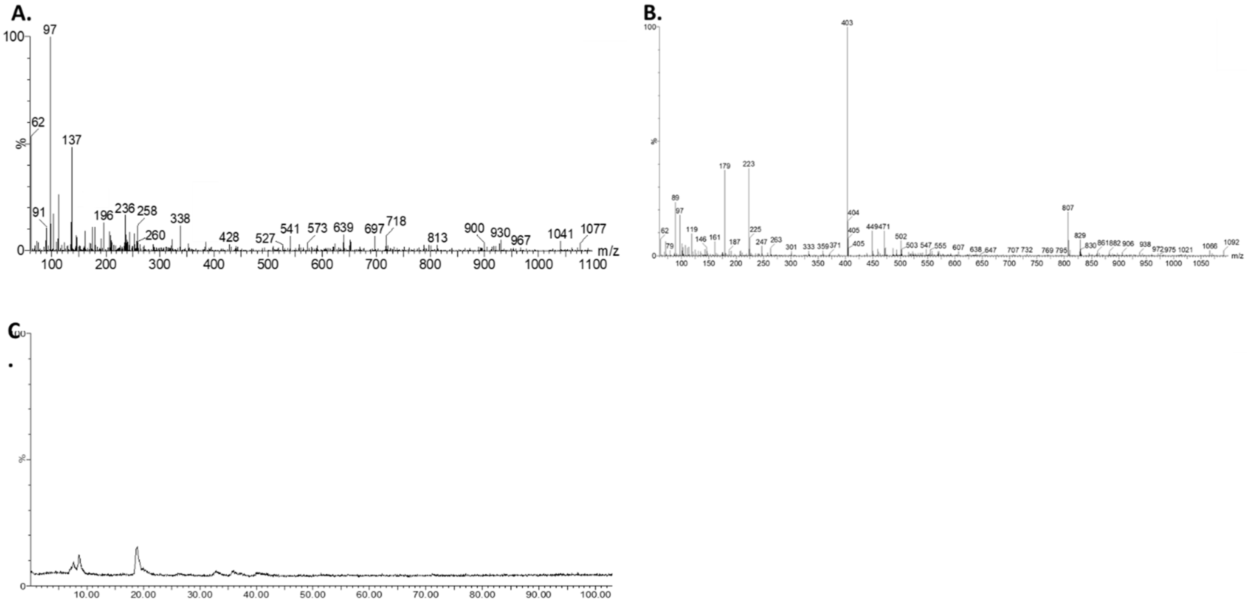

Figure S2. Full scan mass spectra acquired in electrospray ionization source in negative mode (ESI−) from peaks at 15.91 min (**A**) and 18.83 min (**B**) detected in the analysis of the oleuropein (OL) aqueous standard solution after exposure for 2 days at 40 ± 2 °C and 75 ± 5% relative humidity (RH). The scan chromatogram in is also presented (**C**).

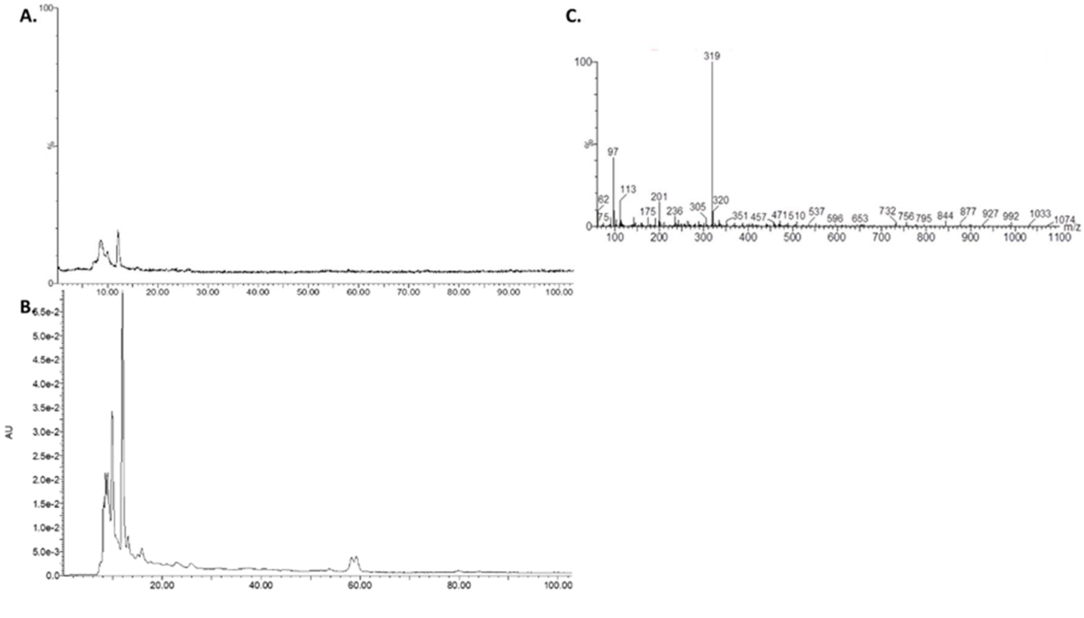

Figure S3. UV Chromatogram at 280nm (**A**), scan chromatogram in electrospray ionization source in negative mode (ESI−) (**B**) and full scan mass spectra from peak at 10.14 min (**C**) detected in the analysis of the hydroxytyrosol (HT) aqueous standard solution after 30 days exposure at 40 ± 2 °C and 75 ± 5% relative humidity (RH).

Table S1. Analysis of variances (ANOVA) of all responses measured for extracts generated by conventional extraction conditions, using olive pomace (OP) stored at different conditions. Significance (p-value<0.05) is presented in bold.

Comparison	p-values							
	AA	TPC	TFC	OL	OLC	HT	TY	EY
**Freeze-dried vs Fresh**	**0.0057**	0.6992	**0.0042**	**0.0175**	**0.0015**	**0.0410**	0.1597	**0.0024**
**Freeze-dried vs De-frozen**	**0.0231**	0.9998	0.9622	**<0.0001**	**0.0052**	0.3166	**0.0302**	0.0623
**Freeze-dried vs Dried**	**0.0002**	0.2495	**<0.0001**	0.5729	**0.0006**	**0.0009**	**<0.0001**	0.1359
**De-frozen vs Fresh**	0.7141	0.7422	**0.0076**	**0.0011**	0.7101	0.4874	0.6505	0.1359
**De-frozen vs Dried**	**0.0161**	0.2762	**<0.0001**	**<0.0001**	0.2582	**0.0079**	**0.0014**	0.9429
**Dried vs Fresh**	0.0712	0.7836	**0.0062**	**0.0033**	0.7870	0.0592	**0.0004**	0.0623
**Freeze-dried vs Fresh**	**0.0057**	0.6992	**0.0042**	**0.0175**	**0.0015**	**0.0410**	0.1597	**0.0024**

*Responses measured: Antioxidant activity (AA), total phenolic content (TPC), total flavonoid content (TFC), extract richness in oleuropein (OL), oleacein (OLC), hydroxytyrosol (HT) and tyrosol (TY), as well as extraction yield (EY)

Table S2. Analysis of variances (ANOVA) of the effect of 2 different defatting methods on the responses measured for extracts generated by conventional extraction conditions, using freeze-dried olive pomace (OP). An extract produced by non-defatted OP was used as the reference. Significance (p-value<0.05) is presented in bold.

Comparison	p-values							
	AA	TPC	TFC	OL	OLC	HT	TY	EY
**Reference vs *n*-hexane**	0.2550	0.4175	0.4327	0.2604	0.9984	>0.9999	0.7684	0.9968
**Reference vs $scCO_2$**	0.4896	**0.0441**	>0.9999	0.9738	0.9935	0.8440	0.6892	0.1816
***n*-hexane vs $scCO_2$**	0.8428	0.2442	0.4327	0.3393	0.9854	0.8440	0.9891	0.1649

*Responses measured: Antioxidant activity (AA), total phenolic content (TPC), total flavonoid content (TFC), extract richness in oleuropein (OL), oleacein (OLC), hydroxytyrosol (HT) and tyrosol (TY), as well as extraction yield (EY)

Table S3. Analysis of variances (ANOVA) of the effect of the extract drying on the stability of the responses measured compared to a freshly obtained liquid extract (reference). Significance (p-value<0.05) is presented in bold.

Comparison	p-values						
	AA	TPC	TFC	OL	OLC	HT	TY
**Reference vs Step 1**	0.6205	0.9791	0.9929	0.9053	0.9665	0.3077	0.9982
**Reference vs Step 2**	**<0.0001**	**0.0170**	0.6559	**0.0122**	0.1711	**0.0027**	**0.0094**
**Step 1 vs Step 2**	**<0.0001**	**0.0212**	0.5920	**0.0078**	0.2342	**0.0132**	**0.0089**

*Responses measured: Antioxidant activity (AA), total phenolic content (TPC), total flavonoid content (TFC), and extract richness in oleuropein (OL), oleacein (OLC), hydroxytyrosol (HT) and tyrosol (TY).

Table S4. Analysis of variances (ANOVA) of the genotoxic effect (alkaline comet assay) of olive pomace (OP) extracts (0.8 mg/mL conventional – CONV and 0.4 mg/mL optimized – OPT3), together with 300 μM (162.2 mg/L) oleuropein (OL), 100 μM (15.4 mg/L) hydroxytyrosol (HT) and their mixture (5 μM/ 2.7 mg/L + 50 μM/7.7 mg/L OL+HT) on human corneal (HCE) and conjunctival (IM-ConjEpi) epithelial cells treated for 24 hours. Treatments are compared to control cells (treated with cell culture medium) and p-values lower than 0.05 were considered statistically significant.

Compared to control	p-values	
	HCE	IM-ConjEpi
**OL**	0.984	0.095
**HT**	1.000	0.982
**OL+HT**	0.450	0.603
**CONV**	1.000	0.359
**OPT3**	0.932	1.000
